# Extracellular matrix ageing as a biological variable in 3D cancer models: A framework for age-aware model design

**DOI:** 10.1016/j.mtbio.2026.103761

**Published:** 2026-10-24

**Authors:** Vanessa Farkas, Anusha Ashokan, Ronja Struck, Hannah O'Neill, Fabio Quondamatteo, Olga Piskareva

**Affiliations:** a Cancer Bioengineering Group, Department of Anatomy and Regenerative Medicine, RCSI University of Medicine and Health Sciences, Dublin, Ireland; b School of Pharmacy and Biomolecular Sciences, RCSI University of Medicine and Health Sciences, Dublin, Ireland; c Tissue Engineering Research Group, Department of Anatomy and Regenerative Medicine, RCSI University of Medicine and Health Sciences, Dublin, Ireland; d Advanced Materials and Bioengineering Research Centre (AMBER), RCSI and Trinity College Dublin, Dublin, Ireland

**Keywords:** Extracellular matrix (ECM) ageing, 3D *in vitro* cancer models, Tumour microenvironment (TME), Mechanobiology, Extracellular matrix/ECM remodelling, Age-aware modelling, Cancer progression

## Abstract

Ageing is a major determinant of cancer risk and outcome, yet age-associated changes in the extracellular matrix (ECM) remain poorly represented in most preclinical cancer models. Throughout life, the ECM undergoes alterations in composition, architecture, biochemical modification, mechanics and turnover that influence cell behaviour and tissue function. Although advanced three-dimensional (3D) cancer models increasingly incorporate ECM components, ageing is rarely considered as an independent biological variable, and the terms aged, ageing-associated and age-mimetic ECM are often applied to biologically distinct experimental systems.

Here, we review current evidence describing ECM remodelling across the lifespan and evaluate how age-associated matrix states have been incorporated into 3D cancer models. We propose a framework that distinguishes four principal modelling strategies: chronologically aged ECM models, defined age-associated ECM modification models, cell-mediated ECM remodelling models, and mixed multicomponent ageing models. These approaches differ substantially in the ageing-associated features they reproduce, the degree of biological fidelity they provide and the strength of mechanistic inference they permit.

Analysis of published studies provides evidence that age-associated ECM states can influence tumour invasion, proliferation, signalling and therapeutic response across many cancer types. At the same time, the available evidence indicates that ECM ageing is a multidimensional process that no single molecular or mechanical surrogate can capture. Hence, we argue that age-aware cancer models should be classified and interpreted according to the specific ageing-associated ECM features they reproduce and experimentally validate. By establishing ECM ageing as a definable biological variable, this framework provides practical guidance for designing, validating, and interpreting next-generation age-aware 3D *in vitro* cancer models.

## Highlights

• ECM ageing is multidimensional and cannot be reduced to matrix stiffness alone.
• Age-aware models fall into four classes: aged, modified, cell-remodelled, mixed.
• Aged ECM alters invasion and drug response; developmental ECM remains untested.
• We propose reporting and validation criteria for age-aware 3D *in vitro* cancer models.

## Graphical abstract

**Figure:**
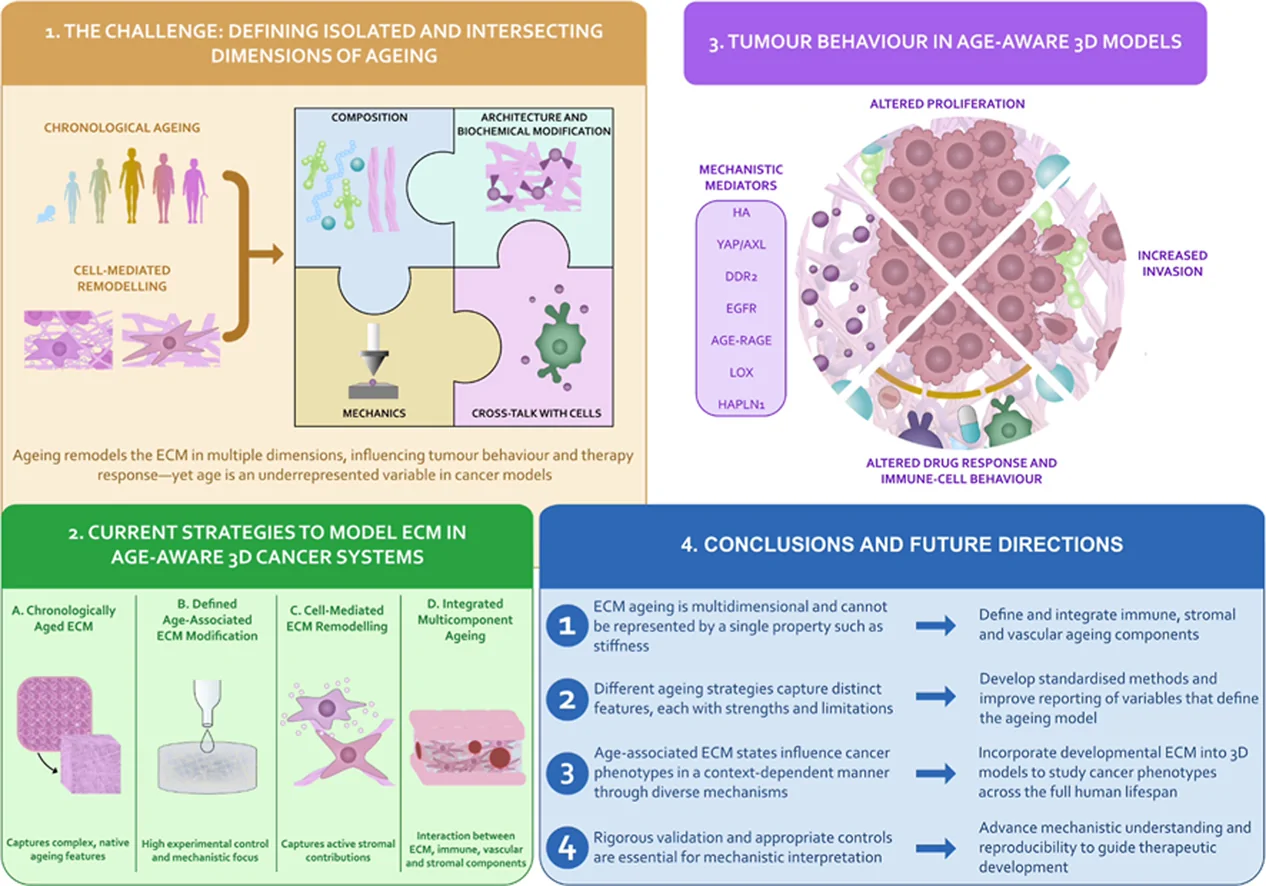

## 1 Introduction

Cancer can arise at any stage of life; however, the majority of diagnoses occur in older adults. In the United States, 60% of new cancer diagnoses are in individuals aged 65 years or older [1], [2]. Paediatric and adolescent cancers are comparatively rare. Of the approximately 2.11 million new cancer cases projected in the United States for 2026, an estimated 15,340 (9680 in children aged 0–14 years and 5660 in adolescents aged 15–19 years) are expected to occur before the age of 20 years [1], [3], [4]. Globally, an estimated 20.6 million new cancer cases occurred in 2024, and population growth and ageing are projected to increase this number by 67%, reaching 34.4 million by 2050 [2]. These trends underscore the importance of understanding how age-associated biological changes influence cancer development, progression, and treatment response.

Tumours are complex tissues in which malignant cells interact dynamically with stromal cells, immune cells, vasculature and the surrounding extracellular matrix (ECM) [5], [6]. Recognising these interplays, along with the need for experimentally tractable and physiologically relevant alternatives to conventional animal models and two-dimensional (2D) culture systems, has driven the development of advanced three-dimensional (3D) *in vitro* cancer models using approaches from regenerative medicine and bioengineering [7], [8], [9], [10], [11].

The ECM is a major structural and regulatory component of the tumour microenvironment (TME). Produced and remodelled by multiple cell populations, including fibroblasts, stromal cells, immune cells and tumour cells, it comprises collagens, several other glycoproteins, proteoglycans, glycosaminoglycans and associated matrix molecules that assemble into tissue-specific supramolecular networks [12], [13]. Beyond providing structural support, the ECM regulates cell adhesion, migration, signalling, tissue organisation, and mechanical behaviour, consequently contributing to both physiological homeostasis and disease progression [6], [14], [15], [16]. Importantly, the ECM does not remain static throughout cancer progression and life (Fig. 1).

**Fig. 1:**
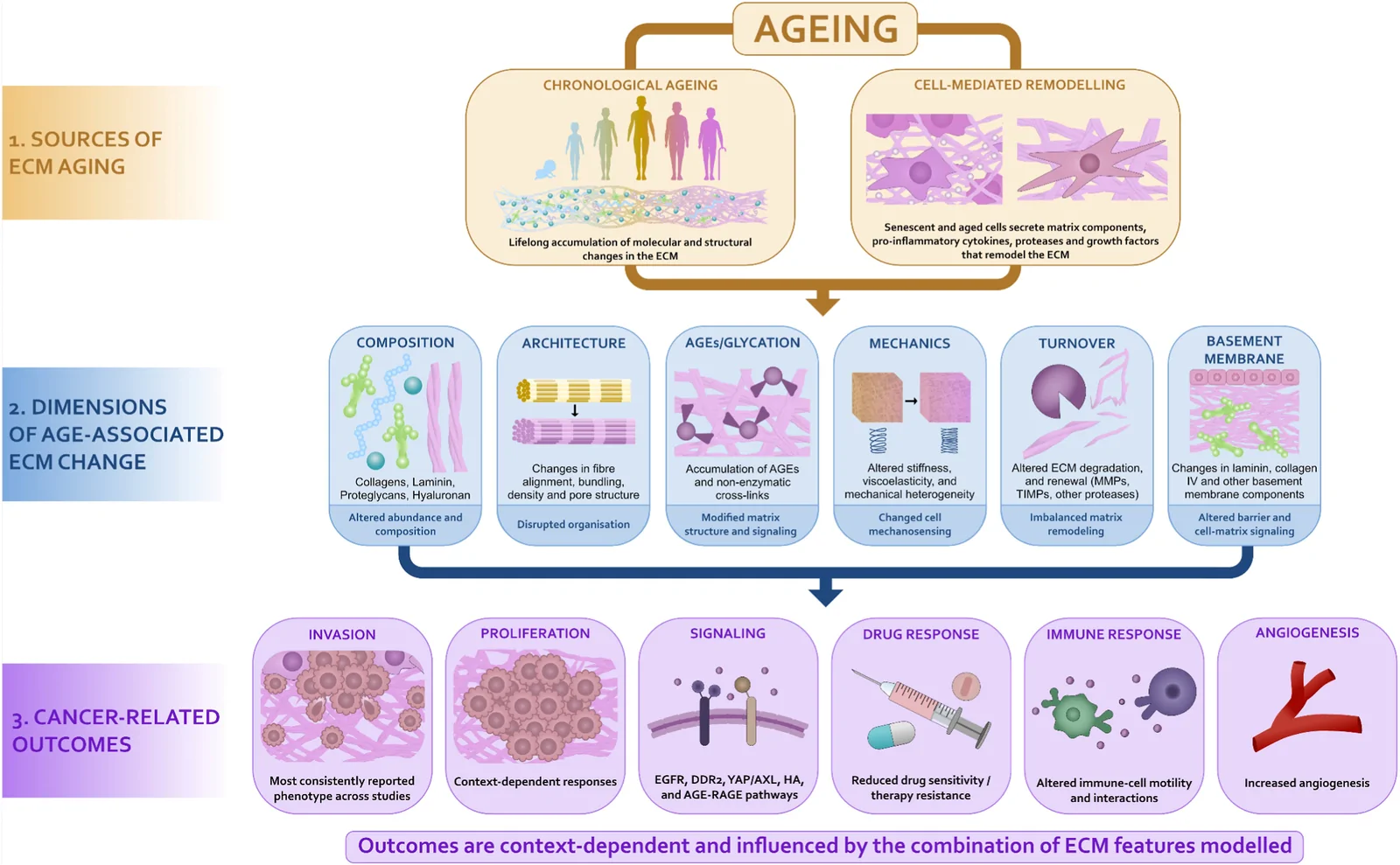
**Sources, dimensions and cancer-related consequences of extracellular matrix ageing.** Chronological ageing and cell-mediated remodelling influence multiple dimensions of ECM biology, including composition, architecture, biochemical modification, mechanics, turnover, basement membrane integrity, immune interactions and cell-mediated remodelling, resulting in context-dependent effects on tumour invasion, proliferation, signalling, therapeutic response, immune interactions and vascular interactions/intravasation.

Despite increasing recognition of the significance of ECM biology in cancer, age remains an underrepresented experimental variable in most preclinical tumour models. Many current 3D *in vitro* systems rely on a single ECM source, donor age, or matrix formulation, and therefore do not capture age-dependent variations in the TME.

Previous reviews have addressed ECM ageing, tumour ECM biology, and advanced 3D *in vitro* cancer models as largely separate topics. However, a conceptual framework for incorporating ECM ageing as an experimental variable within these systems remains lacking. Consequently, studies often use the terms aged, ageing-associated, and age-mimetic ECM interchangeably, despite these representing fundamentally different biological and experimental conditions.

This review synthesises current evidence on age-associated ECM remodelling across the lifespan and evaluates how these changes have been incorporated into 3D *in vitro* cancer models. In addition to summarising the literature, we present a framework that distinguishes chronological ECM ageing, defined age-associated ECM modifications, cell-mediated ECM remodelling, and mixed multicomponent ageing models as complementary modelling strategies. The review further examines how these approaches differ in biological fidelity, mechanistic resolution, and interpretive clarity, and proposes practical design principles for developing and validating next-generation age-aware cancer models.

## 2 Age-associated remodelling of the extracellular matrix

Ageing is accompanied by progressive, tissue-specific alterations in ECM composition, organisation, biochemical modification, turnover and biomechanical behaviour [17], [18], [19], [20], [21]. These changes are not uniform across tissues or life stages; rather, their direction and magnitude depend on native matrix composition, resident and infiltrating cell activity, tissue function, and the mechanical environment. Therefore, ECM ageing should be regarded as a multidimensional process rather than the accumulation of a single molecular or mechanical alteration.

This distinction is particularly important in cancer. Tumour development itself promotes extensive ECM deposition, degradation and reorganisation through interactions among malignant cells, fibroblasts, stromal populations and other components of the TME [6], [13], [15], [16], [22]. Such tumour-associated remodelling may produce matrix features that overlap with those observed during physiological ageing, including modified collagen organisation, increased matrix stiffness and changes in glycosaminoglycan content. However, the underlying biological processes are distinct. Similarly, senescence-associated ECM remodelling represents a cell-mediated component of ageing in which senescent cells alter their surrounding matrix through changes in secretion, degradation and matrix organisation [23], [24].

Thus, an experimentally observed "aged ECM" phenotype requires careful definition of whether the model represents chronological ECM ageing, a specific age-associated matrix modification, or cell-mediated remodelling that also may be associated with senescence.

### 2.1 ECM across the lifespan: developmental, adult and tumour-associated states

ECM states also differ substantially across developmental stages, but paediatric and adult tumours should not be interpreted simply as opposite ends of a chronological ageing continuum (Table 1). Developmental programmes, tissue differentiation, and tumour lineage independently shape ECM composition and organisation and therefore need to be distinguished from ageing-associated remodelling.

**Table 1:** Principal age-associated alterations of the extracellular matrix and their implications for tissue and tumour biology.

ECM feature	Direct evidence for age-associated change	Potential cancer relevance	Current evidence assessment
Collagen	Ageing alters collagen composition (e.g., higher collagen I, VI and XIV in aged human lung), organisation, fibre architecture and cross-linking; aged collagen accumulates AGEs, carboxymethyl-lysine, pentosidine and pyridinoline cross-links [17], [18], [25]	Altered proliferation, invasion, migration, mechanotransduction and therapeutic response have been reported in breast, prostate, lung, melanoma and fibrosarcoma models [25], [26], [27], [28], [29], [30], [31].	Growing evidence links chronological ECM ageing to cancer-related phenotypes, although effects are context dependent rather than uniformly tumour promoting.
Basement membrane (including laminin)	Human retinal basement membrane thickens, loses laminin and stiffens with age; aged mouse lung ECM contains less laminin α3 and α4 [19,37].	May influence epithelial integrity, tumour-cell invasion and tissue architecture. Direct evidence for chronological age-dependent tumour effects remains limited [19], [23], [32].	Strong evidence for age-related alteration; limited direct evidence for cancer-specific consequences of chronological ageing.
Fibronectin	Fibronectin decreases in aged human reticular dermis and in aged mouse lung; senescent fibroblasts assemble fewer fibronectin fibrils [32], [33].	Fibronectin regulates cell adhesion, migration and invasion and is frequently altered during tumour progression; however, direct evidence linking chronological fibronectin ageing to cancer behaviour remains limited [34].	Limited-to-moderate evidence for age-associated remodelling; insufficient direct evidence for cancer-specific consequences of chronological fibronectin ageing.
Hyaluronan (HA)	Total HA content and polymer size are stable in ageing human skin, but tissue-bound HA increases [35]; aged tail-tendon collagen contains about twice as much HA as young collagen [28], [36].	Altered HA signalling may influence inflammation, proliferation and tumour behaviour. Increased HA within aged collagen matrices has been linked to enhanced prostate epithelial-cell proliferation [28], and low-molecular-weight HA induces an inflammatory response in ovarian stromal cells [37], [38].	Moderate evidence supporting a role in age-associated TME remodelling.
ECM architecture	Ageing alters fibre alignment, bundling, density, fragmentation, pore structure and network organisation [26], [29], [30], [31], [39].	Changes in architecture influence cell migration, invasion, mechanotransduction and immune-cell trafficking [26], [30].	Strong evidence that matrix architecture is a biologically important feature of ECM ageing and should be evaluated independently of bulk stiffness.
Biochemical modification (AGEs/glycation)	Ageing promotes accumulation of advanced glycation end products and collagen cross-links, altering matrix structure and function [18].	Glycated matrices affect tumour-cell morphology, migration and signalling, including AGE-RAGE-mediated pathways [40].	Moderate–high mechanistic evidence for a defined age-associated ECM modification, supported by functional interrogation of AGE–RAGE signalling; however, glycation does not reproduce the entire ageing phenotype.
Mechanical properties	Age-associated changes in stiffness, viscoelasticity and mechanical heterogeneity have been reported across multiple tissues [19], [21], [26], [29].	Matrix mechanics regulate proliferation, migration, invasion and therapy response through mechanotransduction pathways [41], [42].	Strong evidence for biological relevance; however, ECM ageing and tumour progression cannot be reduced to a universal “stiffer matrix” model.
Cell-mediated ECM remodelling	Ageing and cellular senescence alter ECM turnover through changes in matrix-remodelling enzymes and stromal-cell activity [20], [23], [24].	Altered matrix turnover contributes to invasion, tissue remodelling and tumour-stromal signalling [43].	Mechanistically important, but distinct from intrinsic ageing of the acellular ECM.

Paediatric solid tumours, including neuroblastoma, rhabdomyosarcoma, Ewing sarcoma and osteosarcoma, contain diverse ECM components, including collagens, tenascin C, vitronectin and proteoglycans [44], [45], [46], [47], [48], [49], [50], [51]. Increased collagen density promotes rhabdomyosarcoma invasion through YAP–PIEZO1 signalling [44], while Wnt/β-catenin activation enhances the secretion of collagens and tenascin C by Ewing sarcoma cells [46]. In patient cohorts, collagen XVIII expression in rhabdomyosarcoma [45], stromal collagen XI in neuroblastoma [48] and reticulin fibre organisation in high-risk neuroblastoma [49] are associated with poorer outcomes. Conversely, vitronectin has been linked to both neuroblastoma differentiation [51] and to high-risk tumour features [50]. In certain tumour types, including rhabdomyosarcoma, tumour cells themselves contribute substantially to ECM production, highlighting the heterogeneity of the paediatric TME [52]. Neuroblastoma studies further demonstrate that tumour-cell phenotype and invasion strategy depend strongly on ECM context, emphasising the biological relevance of matrix composition in early-life cancers [53]. These findings indicate that age-related modelling should account for biological differences between developmental and ageing tissues, rather than treating paediatric ECM as simply an "unaged" version of adult ECM.

Adult epithelial tumours commonly exhibit extensive tumour-associated remodelling characterised by deposition of collagen, fibronectin, hyaluronan and matricellular proteins, often accompanied by fibroblast activation and desmoplastic tissue formation [6], [15], [16], [22]. These alterations are primarily associated with tumour progression and cannot be used in isolation as markers of physiological ECM ageing.

Comparative analyses further show heterogeneity in ECM states across the lifespan and between tumour types. Paediatric and adult high-grade gliomas, for example, share comparable glycosaminoglycan deposition and partially overlapping ECM proteomes, whereas paediatric tumours have lower, less variable collagen deposition [54]. Pan-cancer analyses likewise show that matrisome gene expression profiles distinguish tumour types and that matrisome genes are frequently affected by copy-number alterations and mutations, with patterns that differ between cancer types [55]. Hence, an age-aware model must define both the age variable and the tissue or tumour context under investigation.

### 2.2 Chronological ECM ageing

Direct comparisons between young and aged tissues provide the closest evidence for chronological ECM remodelling. Across multiple organs, ageing has been associated with alterations in collagen fibril organisation, basement membrane composition, glycosaminoglycan content and matrix-remodelling activity [17], [19], [20], [32].

Collagen is among the most extensively studied components of the ageing ECM. Because fibrillar collagens are relatively long-lived proteins, they accumulate structural and biochemical modifications over time, including altered cross-linking and non-enzymatic glycation [18], [25]. These modifications can affect fibril organisation, matrix architecture and cell–matrix signalling. Studies using young- and aged-derived collagenous matrices further indicate that age-associated matrix states can alter cellular behaviour, although the magnitude and direction of these effects vary across experimental systems [25], [26], [27], [28], [29], [30].

Ageing also affects basement-membrane components. The inner limiting membrane of the human retina, for example, thickens from approximately 70 nm at fetal stages to several micrometres by age 90, loses laminin relative to collagen IV and agrin, and becomes stiffer with age [19]. Similarly, decellularised lung scaffolds from 1-year-old mice contain less laminin α3 and α4, elastin and fibronectin, and more collagen than scaffolds from 3-week-old mice, and reduce laminin expression by seeded human lung epithelial cells and fibroblasts [32]; this comparison spans the transition from juvenile to adult tissue rather than old age. Hyaluronan (HA) also changes with age, although not uniformly [36]. In a small autopsy series of human skin (two donors each at fetal, adult and elderly ages), total HA content and polymer size did not differ with age, but the tightly tissue-bound fraction increased from 7% of total HA in fetal skin to 23% in elderly skin [35], whereas collagen extracted from aged mouse tail tendons contains approximately twice as much HA as young collagen*,* and HA re-introduction increased the proliferation of benign prostate epithelial cells cultured on young collagen gels [28].

These findings demonstrate that chronological ECM ageing can involve concurrent changes across multiple matrix components rather than modification of a single dominant molecule. This distinction provides an important biological basis for evaluating whether an experimental model genuinely represents an aged ECM or merely reproduces one selected feature of ageing.

### 2.3 Structural, biochemical and mechanical remodelling

In addition to changes in the ECM composition, ageing alters its higher-order organisation. Collagen fibrils can become fragmented, reoriented, more highly cross-linked or differently organised within the tissue microenvironment [18], [20]. Such changes can modify pore architecture, ligand presentation, matrix permeability and cell–matrix interactions without necessarily being reflected by changes in bulk composition.

Biochemical modification represents another important dimension of ECM ageing. One well-characterised mechanism is the accumulation of advanced glycation end products (AGEs), which arise through non-enzymatic reactions between reducing sugars and long-lived matrix proteins [18]. AGE accumulation promotes covalent modification and cross-linking of matrix proteins and can consequently alter matrix structure, mechanical behaviour and receptor-mediated signalling. Experimental studies using glycated collagen further demonstrate effects on tumour-cell morphology, migration and AGE–RAGE-associated signalling, supporting the functional relevance of this biochemical modification in cancer models [40].

Mechanical properties are closely coupled to these biochemical and structural changes. Multiple tissues show age-associated alterations in stiffness, viscoelasticity, and mechanical heterogeneity [19], [26], [29]. However, these changes do not follow a universal trajectory. Matrix mechanics depend on composition, cross-linking, hydration, fibre organisation, and the scale and method of measurement [21]. Accordingly, increased stiffness should be regarded as one possible component of ECM ageing rather than as a general surrogate for an aged matrix.

This distinction is particularly important in cancer biology. Matrix mechanics can regulate proliferation, migration, invasion and therapeutic response through mechanotransduction pathways [41], [42]. Nevertheless, similar phenotypes may also result from changes in ligand availability, matrix architecture, biochemical modification or receptor-mediated signalling. Therefore, mechanobiological observations should be interpreted within the more extensive biochemical and structural context of the ECM rather than attributed to stiffness alone.

### 2.4 ECM turnover and cell-mediated remodelling

ECM ageing also involves changes in matrix turnover. Matrix metalloproteinases (MMPs) and related proteases regulate ECM degradation and renewal and have established roles in both tissue homeostasis and cancer progression [43]. Altered expression of matrix-remodelling enzymes in aged tissues and fibroblasts suggests that dysregulated turnover contributes to age-associated changes in matrix composition and organisation [20].

Cellular senescence provides another mechanism of cell-mediated remodelling by which ageing can modify the extracellular environment. Senescent stromal cells secrete cytokines, growth factors and matrix-remodelling enzymes collectively associated with the senescence-associated secretory phenotype (SASP) [24]. These factors can modify collagen deposition, matrix degradation and ECM organisation and, thereby, alter the behaviour of neighbouring epithelial and tumour cells [24]. Senescence can also alter the matrix produced by epithelial cells themselves: induction of the senescence-associated gene mac25 in prostate cancer cells increased laminin α4 and β2 chain expression, and these chains altered prostate cancer cell migration and tumorigenicity *in vivo* [23]. Importantly, senescence-associated ECM remodelling should not be considered synonymous with chronological ECM ageing. A senescence-based model combines ECM remodelling with active cellular signalling and thus represents an ageing-associated cellular microenvironment rather than an isolated aged acellular matrix. This distinction becomes particularly important when experimental studies seek to assign a tumour phenotype specifically to ECM-intrinsic ageing.

### 2.5 Defining the modelling dimensions of ECM ageing

ECM ageing involves interconnected changes in composition, architecture, biochemical modification, mechanics and turnover that may occur concurrently during chronological ageing but can be reproduced selectively in experimental systems. Consequently, age-aware models should be evaluated according to which ageing-associated dimensions they reproduce, how closely these reflect naturally occurring ageing, and the degree of mechanistic control they provide. This distinction underpins the experimental framework considered in Section 3.

## 3 Experimental strategies for modelling ECM ageing

Experimental modelling of ECM ageing presents considerable challenges. Chronological ageing is a lifelong biological process that involves the simultaneous evolution of ECM composition, architecture, biochemistry, mechanics, cellular interactions, and systemic influences. Experimental systems, by necessity, simplify this complexity by isolating tractable and controllable variables.

As a result, the term 'aged ECM' encompasses several distinct experimental strategies. Some models replicate naturally accumulated age-associated changes using chronologically aged tissues, while others introduce specific ageing-associated features into otherwise controlled matrices. Additional approaches incorporate cellular processes, such as senescence-driven remodelling. Although all of these models are considered age-aware, they address different biological questions and allow for varying degrees of mechanistic inference.

Age-aware ECM cancer models can be classified into four methodological categories: (i) chronologically aged ECM models, (ii) defined age-associated ECM modification models, (iii) cell-mediated ECM remodelling models, and (iv) mixed multicomponent ageing models. These categories represent complementary strategies along a continuum between biological fidelity and experimental control, rather than competing approaches.

Model selection within this framework should be guided by the specific experimental question. High-fidelity models are optimal for determining whether naturally aged matrices influence tumour behaviour, whereas reductionist systems are better suited to identifying causal mechanisms. More complex multicellular models facilitate investigation of age-associated interactions among ECM, stromal, vascular, and immune components. Table 2 summarises the principal capabilities and limitations of these modelling approaches.

**Table 2:** Experimental representation of age-associated ECM dimensions: capabilities and limitations.

Age-associated dimension	Experimental representation	What can be modelled	Principal limitation
**ECM composition**	Chronologically aged ECM; decellularised tissues; tissue-derived hydrogels; isolated ECM components	Native age-associated differences in matrix proteins, glycosaminoglycans and other ECM constituents	Multiple components change concurrently, limiting attribution to individual molecules
**Biochemical modification**	Glycation/AGE formation; oxidative modification; chemical or enzymatic cross-linking	Defined molecular modifications associated with ageing and their effects on matrix function	Represents selected modifications rather than the cumulative biochemical history of ageing
**Fibre architecture**	Decellularised ECM; collagen-based matrices; engineered and biofabricated scaffolds	Fibre density, alignment, organisation, fragmentation and pore structure	Native spatial heterogeneity and tissue-specific organisation are difficult to reproduce
**Mechanical properties**	Tunable hydrogels; controlled cross-linking; engineered matrices	Selected changes in stiffness, viscoelasticity and mechanical loading	Mechanical alteration alone does not reproduce the biochemical and structural complexity of ECM ageing
**ECM turnover**	Protease-mediated remodelling; cell-mediated deposition and degradation	Selected aspects of matrix degradation, deposition and remodelling dynamics	Lifelong turnover and cumulative remodelling history cannot readily be reproduced
**Cell-mediated remodelling**	Senescent fibroblasts or mesenchymal stromal cells; stromal–tumour co-culture	Age-associated changes in ECM deposition, degradation and organisation together with cellular signalling	ECM-dependent effects cannot always be separated from SASP-mediated paracrine effects
**Inflammatory/immune interactions**	Cytokine exposure; immune–stromal co-culture; multicellular systems	Selected inflammatory signals and immune–ECM interactions	Systemic inflammageing and the full diversity of immune ageing remain difficult to reproduce
**Vascular and stromal interactions**	Organotypic cultures; microfluidic and organ-on-chip systems; multicellular 3D models	Interactions between ECM, stromal, vascular and immune components	Increasing complexity introduces additional variables and requires extensive validation
**Chronological ageing**	Young versus naturally aged tissues or ECM	Integrated age-associated changes accumulated *in vivo*	Age, donor variability and tissue history cannot readily be separated; causal ECM features remain difficult to identify
**Integrated ageing phenotype**	Combination of aged ECM, defined modifications and/or aged or senescent cells	Interactions among multiple ageing-associated ECM and cellular features	No current model reproduces the full temporal, spatial and systemic history of ECM ageing

### 3.1 Chronologically aged extracellular matrices: biological fidelity with limited mechanistic control

The most direct approach is to compare ECM derived from young and aged tissues. These models include decellularised matrices (dECM), tissue-derived hydrogels and isolated ECM components such as collagen [25], [26], [27], [28], [29], [31], [32], [39], [56], [57].

The primary strength of these models is that age-associated changes develop naturally during tissue ageing, rather than being imposed experimentally. Consequently, chronologically aged matrices retain combinations of changes in composition, fibre architecture, cross-linking, and other properties that are difficult to reproduce independently [26], [39], [57].

These models serve as a critical reference state for assessing the biological relevance of age-associated ECM changes to tumour behaviour.

However, this biological fidelity limits mechanistic resolution. Multiple ECM variables differ between young and aged matrices, complicating attribution of observed tumour phenotypes to specific features. Donor age, sex, tissue source, anatomical location, and processing methods introduce additional variability. Decellularisation can alter ECM properties and requires validation. For example, decellularisation and delipidation increased the elastic modulus of aged mouse breast tissue 4.3-fold, but only 1.3-fold in young tissue [26].

Chronologically aged ECM should be regarded as a high-fidelity reference model rather than a mechanistically comprehensive system. Its primary value lies in determining whether the naturally aged extracellular environment produces a distinct phenotype. Subsequent mechanistic investigations can then employ more controlled age-mimetic systems.

### 3.2 Defined age-associated ECM modifications: experimental control with reduced biological completeness

Defined age-associated ECM modifications represent the converse experimental strategy. Instead of transferring the complete aged matrix state, researchers introduce a specific ageing-associated feature into an otherwise controlled matrix.

Collagen glycation is a prominent example. Ageing promotes the accumulation of AGEs in long-lived ECM proteins, and researchers can experimentally induce glycation in collagen matrices to reproduce selected biochemical and structural consequences of this process [18]. For example, glucose-induced glycation of collagen I causes fibre bundling and increases pore size and matrix stiffness [40].

Related reductionist approaches include cross-linking by reactive dicarbonyls such as methylglyoxal, which cross-links collagen I and degrades laminin-rich basement membrane [58], and controlled alteration of matrix mechanics, including progressive stiffening of hydrogels over time [10], [59], [60]. In one such model, stiffening of an adipose ECM–silk fibroin hydrogel by 10–15 kPa over three weeks induced growth arrest and phenotypic heterogeneity in MCF-7 breast cancer cells [59], showing that increased stiffness alone need not promote tumour growth.

The principal advantage of these systems is mechanistic precision. Researchers can prepare young and modified matrices from the same base material while experimentally altering a defined variable. This approach enables testing whether a specific ageing-associated modification is sufficient to induce a cellular or tumour phenotype.

However, such models should be described as models of specific ageing-associated mechanisms rather than comprehensive models of chronological ECM ageing. For example, glycation may alter cross-linking, architecture, and receptor signalling, but it does not reproduce the simultaneous changes in matrix composition, turnover, fibre organisation, and cellular activity that accumulate throughout life. Similarly, mechanically stiffening an otherwise unchanged hydrogel isolates the effect of mechanics but does not replicate the biochemical and structural history underlying mechanical changes in native tissue. Hybrid scaffolds offer an intermediate approach. Decellularised cardiac ECM from young (1–2 months) or aged (18–24 months) mice has been combined with synthetic hydrogels tuned to young or aged tissue stiffness (approximately 13 and 39 kPa), demonstrating that the ligand presentation of young ECM can outweigh aged stiffness in maintaining cardiac fibroblast quiescence [57].

This distinction is essential for interpreting age-aware three-dimensional models. Although experimental control reduces complexity, it should not be equated with greater physiological completeness.

### 3.3 Cell-mediated ECM remodelling: modelling ageing as an active cellular process

A third strategy introduces cellular activity into the ageing model. Cell-mediated ECM remodelling (CMR) encompasses models in which living cells actively alter the ECM through matrix deposition, degradation, crosslinking, organisation, or other remodelling processes. In this broad category, the cellular state responsible for matrix modification may be experimentally defined without necessarily being senescent. For example, Kaur et al. used fibroblasts from donors of different ages, providing a model in which age-associated differences in fibroblast activity contribute to ECM remodelling [30]. These models capture the dynamic nature of ECM ageing but do not isolate a single senescence-associated mechanism.

Senescence-associated ECM remodelling (SAM) represents a more specific form of cell-mediated remodelling in which cellular senescence is an experimentally defined driver of ECM alteration. Senescent fibroblasts, mesenchymal stromal cells, and related populations can modify ECM composition, organisation, and turnover through altered matrix deposition, degradation, and senescence-associated secretory signalling [24], [61], [62]. SAM models therefore incorporate both matrix remodelling and the signalling activity of senescent cells, making them valuable for investigating how cellular ageing contributes to the extracellular environment. However, this also means that ECM-dependent effects may be difficult to distinguish from soluble or cell-contact-mediated effects generated by the senescent population. Appropriate experimental controls are therefore required to distinguish matrix-dependent effects from direct paracrine effects of senescent cells.

The methodological advantage of this approach is its capacity to capture a key property of ageing that acellular systems cannot readily reproduce: the continuous generation and remodelling of the aged microenvironment by cells. These models can thus reproduce changes in matrix composition and architecture while simultaneously incorporating cell-derived signalling.

### 3.4 Dynamic and multicomponent models: towards a more complete ageing microenvironment

The limitations of reductionist systems have motivated the development of approaches that combine multiple ageing-associated variables. Emerging platforms enable integration of age-associated ECM states with stromal, immune, or vascular components using biofabrication, microfluidics, and organ-on-chip technologies to control spatial and temporal interactions [9], [63], [64], [65].

These systems can reproduce interactions that conventional ECM-only models cannot readily capture. For example, age-associated changes in matrix mechanics may influence stromal cell behaviour, while stromal cells can subsequently modify matrix composition and immune cell migration. Incorporating multiple cellular components may thus reveal emergent phenotypes not apparent when each component is studied in isolation.

Nevertheless, increased complexity does not necessarily equate to greater validity. Each additional component introduces new variables, and a complex model becomes difficult to interpret if the intended ageing phenotype is not independently verified. Multicomponent models should be accompanied by systematic characterisation of ECM composition, architecture, and mechanics and, where relevant, cellular senescence, inflammatory state, and immune or vascular function.

The most informative strategy may consequently be modular rather than maximalist: validate individual components independently first, then combine them when the biological question requires their interaction.

### 3.5 Guidelines for selecting and validating age-aware models

No single experimental system can replicate all aspects of ECM ageing. Model selection should therefore be guided by the specific biological question, with a clear distinction between biological fidelity, defined as the extent to which a model reproduces naturally occurring age-associated ECM features, and mechanistic resolution, defined as the extent to which individual determinants can be isolated and causally tested.

Three complementary questions can guide model selection. First, is an age-associated ECM state itself relevant to the phenotype of interest? Chronologically aged matrices best address this question because they retain naturally accumulated age-associated features, although multiple concurrent changes can limit attribution to individual determinants. Second, which ECM feature contributes to the observed phenotype? Defined modification models provide greater mechanistic resolution and can test whether a specific ageing-associated feature is sufficient to alter cellular behaviour. Third, does the identified mechanism remain operative within a more complex ageing microenvironment? Senescence-associated and integrated multicomponent models can address interactions between ECM remodelling and stromal, immune, or vascular components.

Regardless of model type, four criteria are essential for interpretation. The ageing variable must be explicitly defined; the resulting ECM phenotype should be experimentally validated rather than inferred solely from donor age or treatment condition; relevant biochemical, structural, and mechanical properties should be characterised independently; and model complexity should remain proportionate to the biological question. These considerations enable age-aware models to be interpreted in terms of both biological relevance and mechanistic insight.

## 4 Evidence from age-aware 3D cancer models

To differentiate age-associated ECM models from the broader field of cancer biology, we conducted a PubMed search using the keywords ‘extracellular matrix/ECM’, ‘ageing’, ‘3D *in vitro* cancer models’, and ‘extracellular matrix/ECM remodelling’. We limited the search to original research articles published in English.

The search covered articles published between 1 January 1980 and 1 July 2026. After screening titles and abstracts, we shortlisted 26 papers for full-text review and included 11 relevant primary studies. The studies summarised in Table 3 were selected based on these criteria: (i) use of a three-dimensional cancer model with cells embedded in or cultured on a 3D matrix, (ii) incorporation of a chronologically aged ECM, a defined age-associated ECM modification, or a cell-mediated ECM remodelling strategy, and (iii) assessment of a measurable cancer-related outcome. Studies without an explicit age-associated ECM component were considered elsewhere when relevant but excluded from this focused evidence synthesis.

**Table 3:** Experimental models investigating age-associated extracellular matrix remodelling in 3D *in vitro* cancer systems.

Study	Evidence class	Cancer model and 3D format	Young/control vs aged/modified ECM	ECM validation	Mechanical characterisation (young vs aged)	Cancer outcome	Mechanistic interrogation
Bahcecioglu et al. [26]	CA	KTB21 (non-malignant mammary epithelial) and MDA-MB-231 breast cancer; cells seeded on dECM (±Matrigel); spheroid and transwell assays	Mouse breast dECM; young 3–6 vs aged 20–23 months	SHG: thinner, curlier fibres in aged ECM; proteomics: reduced collagen V and XV, increased inflammatory proteins	Nanoindentation. Native tissue 509 ± 275 vs 356 ± 162 Pa (aged softer); decellularised 1180 ± 1226 vs 1867 ± 1765 Pa (aged stiffer)	Increased motility and invasion; E-cadherin delocalisation and EMT-like gene expression in KTB21	LOX siRNA in the seeded cells reverted aged-matrix phenotypes (invasion also reduced on young matrix)
Yang et al. [39]	CA	MDA-MB-231 spheroids embedded in collagen; KTB21 and mammary fibroblasts for proliferation and ageing markers	Mouse tail-tendon collagen I (3 mg/mL); young 2–6 vs aged 20–24 months	Aged fibres thicker; similar pore size; higher lectin binding (SNA-I, WGA, PHA-E); >90% collagen retained over 7 days	Rheology G′ 20.2 ± 1.6 vs 19.0 ± 1.7 Pa; nanoindentation E < 80 Pa in both (no difference)	Increased motility and invasion; proliferation unchanged. Screen of 720 FDA-approved drugs (10 μM): 36 effective in both, 27 in young only, 7 more effective in aged	None
Bianchi-Frias et al. [27]	CA	TRAMP-C2 and Myc-CaP mouse prostate cancer; cells seeded on collagen gels	Mouse tail-tendon collagen (0.6 mg/mL); young 4 vs aged 20–24 months	Not characterised (collagen content normalised by Sircol assay)	Not measured	Invading cells, aged vs young: 17.2 ± 3.9 vs 0 (TRAMP-C2) and 153.5 vs 2.8 (Myc-CaP); increased TRAMP-C2 proliferation	None *in vitro* (*in vivo*: faster tumour growth and more macrophages in aged hosts)
Damodarasamy et al. [28]	CA	PEC (normal), BPH-1 (benign) and LNCaP (prostate cancer); cells seeded on collagen gels	Mouse tail-tendon collagen; young 5–6 vs aged 23–24 months	HA 8.0 ± 1.6 vs 3.9 ± 2.4 ng/μg collagen (aged vs young); similar HA molecular weight	Not measured	Increased proliferation of all three lines on aged collagen	HA supplement to young collagen increased proliferation of BPH-1 only (not PEC or LNCaP)
Sarazin et al. [31]	CA	A549 lung carcinoma and BZR transformed bronchial epithelial cells; embedded in collagen (3D) vs collagen coating (2D)	Rat tail-tendon collagen I; adult 10 vs old 100 weeks	SHG: shorter, thinner fibres in old collagen	Not measured	3D only: erlotinib IC50 18 → 26 μM (A549) and 15 → 21 μM (BZR); increased A549 growth. No differences in 2D	Associative: EGFR expression (∼1.5-fold) and phosphorylation (∼1.5–2-fold) higher in old collagen; not perturbed
Saby et al. [25]	CA	HT-1080 fibrosarcoma (A204 sarcoma confirmatory); embedded in collagen (3D) vs coating (2D)	Rat tail-tendon collagen I; adult 2 months vs old 2 years	Higher fluorescent AGEs, CML and pentosidine (LC-MS/MS) and HP/LP cross-links in old collagen	Not measured	Loss of adult-collagen growth suppression; 3D only	Reduced DDR2/SHP-2 activation, increased JAK2/ERK, reduced p21; DDR2 inhibition phenocopied old collagen; RAGE not involved (dominant-negative RAGE)
Florent et al. [29]	CA	1205Lu and SKMEL28 BRAF^V600E^ melanoma; embedded cells and spheroids; xenografts in collagen	Rat tail-tendon collagen I; young 2 months vs old 2 years	CT-FIRE: shorter, thinner, straighter fibres in old collagen	ElastoSens Bio: G′ 1553 ± 155 vs 2815 ± 487 Pa (old stiffer)	1205Lu: increased proliferation and invasion; SKMEL28: proliferation unchanged. Vemurafenib IC50 in 3D 3.79 → 6.76 μM (1205Lu) and 0.9 → 1.8 μM (SKMEL28); vemurafenib ineffective in xenografts in old collagen	Increased YAP nuclear localisation (associative); AXL inhibition reduced invasion in young and old collagen; GAS6 increased invasion only in old collagen
Dandia et al. [40]	AAM	MDA-MB-231 breast cancer; cells seeded on gel surface (morphology, migration) and 3D invasion assay	Collagen I: unglycated control vs glycated with 50 or 500 mM glucose (ribose in selected experiments)	Glycation confirmed (FTIR, circular dichroism, calorimetry); fibre bundling; increased pore size	Rheology: storage modulus increased with glucose concentration	Altered morphology and increased migration; viability and proliferation unchanged	Anti-RAGE antibody attenuated glycation-associated responses
Kumar et al. [58]	Mixed (AAM + CMR)	MDA-MB-231 breast cancer in collagen I (1 mg/mL) in the tumour chamber of a microfluidic chip, adjacent to a perfused TeloHAEC endothelial channel on laminin-rich basement membrane	Untreated vs 200 μM methylglyoxal (endothelial pre-exposure and perfusion medium); cell-free collagen I and lrBM gels treated with 50–200 μM methylglyoxal for 1–4 days	Collagen I: reduced picrosirius red staining, attenuated fibrillar appearance (SEM), SHG and FTIR evidence of cross-linking; lrBM: progressive dissolution	Not measured	Increased cancer-cell intravasation into the vascular channel by day 7 (p = 0.008); reduced adhesion and spreading of cancer and endothelial cells on treated matrices	Methylglyoxal also induced endothelial senescence and shedding; matrix and endothelial contributions not separated
Ghosh et al. [61]	CMR (SAM)	MDA-MB-231 co-spheroids with MSCs, embedded in collagen	Collagen I (1.5 mg/mL) with co-embedded senescent (15 Gy γ-irradiation) vs pre-senescent bone-marrow MSCs from a 24-year-old donor	Collagen density, fibre width and alignment; ECM gene expression (COL1, COL6, LOX, TGM2, POSTN)	Bulk ECM mechanics not measured (intracellular particle tracking only)	Increased spheroid growth and invasion with senescent MSCs	MMP inhibitor (GM6001) blocked invasion in both conditions; LOX inhibitor (BAPN) and anti-periostin partially reduced senescence-associated invasion (not significant for single-cell invasion)
Kaur et al. [30]	Mixed (CA + CMR)	1205Lu and WM3918 melanoma in 3D skin reconstructs and fibroblast-derived matrices; Yumm1.7 in young vs aged mice	Matrix deposited by human dermal fibroblasts from young (<45 years) vs aged (>55 years) donors	Collagen fibre alignment and organisation; loss of HAPLN1 from aged fibroblast secretome	Not measured (fibre-network mechanics modelled computationally)	Increased melanoma invasion and metastasis; altered immune-cell motility in aged matrix	HAPLN1 knockdown (young) and recombinant HAPLN1 (aged) reciprocally modulated matrix organisation and invasion

**Evidence classes.** CA, chronologically aged ECM compared between young and aged sources; AAM, defined age-associated modification applied to an otherwise identical matrix; CMR, cell-mediated ECM remodelling by living cells; SAM, senescence-associated remodelling, a subtype of CMR in which senescence is the experimentally defined driver; Mixed, donor chronological age coupled to cell-mediated remodelling.

**ECM validation** refers to experimental characterisation of the intended age-associated ECM phenotype (composition, architecture or biochemical modification).

**Mechanical characterisation** refers to direct measurement of matrix mechanics; values are young (or control) vs aged (or modified), mean ± SD as reported. “Not measured” indicates that no matrix mechanical measurement was reported.

**Cancer model.** Malignant status is stated; KTB21, PEC and BPH-1 are non-malignant. “Seeded on” indicates culture on the surface of a 3D matrix rather than embedding within it.

Abbreviations: AGEs, advanced glycation end products; BAPN, β-aminopropionitrile; CML, Nε-(carboxymethyl)lysine; dECM, decellularised ECM; E, elastic modulus; G′, storage modulus; HA, hyaluronan; HP/LP, hydroxylysyl-/lysylpyridinoline; lrBM, laminin-rich basement membrane; MSC, mesenchymal stromal cell; SHG, second harmonic generation.

### 4.1 Chronologically aged extracellular matrices

Chronologically aged extracellular matrices provide the closest experimental representation of age-associated ECM states because they retain changes accumulated during tissue ageing. Across multiple cancer types, these models provide evidence that age-associated ECM states can alter cellular behaviour, including invasion, proliferation, and therapeutic response [25], [26], [27], [29], [31], [39].

One clear example comes from Bahcecioglu et al., who compared breast dECM derived from young and aged mice. After processing, the aged decellularised matrix was stiffer than the young matrix, even though native aged breast tissue was softer. It promoted motility, invasion, and EMT-like changes in non-malignant KTB21 mammary epithelial cells and MDA-MB-231 cells. siRNA knockdown of LOX in the seeded cells reverted these changes, implicating cell-derived LOX in the response to the aged matrix [26]. In prostate models, collagen extracted from aged mouse tail tendons increased proliferation of prostate epithelial and cancer cells and invasion of prostate cancer cells [27], [28]. Aged collagen contained about twice as much hyaluronan as young collagen, and hyaluronan supplement increased proliferation of benign BPH-1 cells but not LNCaP cancer cells [28]. Yang et al. observed enhanced invasion of breast cancer spheroids in aged tail-tendon collagen despite near-identical bulk mechanics (storage modulus about 20 Pa in both) and no change in proliferation. In a screen of 720 FDA-approved drugs, 27 were effective only in young and 7 performed better in aged matrices [39].

Importantly, aged ECM effects were not limited to tumour growth and invasion. Sarazin et al. showed that old collagen increased EGFR expression and phosphorylation and raised the erlotinib IC50 of A549 lung carcinoma and BZR transformed bronchial cells by about 40% in 3D, but not 2D, culture [31]. Florent et al. reported about a two-fold increase in the vemurafenib IC50 of two BRAFV600E melanoma cell lines in old collagen, which was also substantially stiffer than young collagen (storage modulus 2.8 vs 1.6 kPa), and loss of vemurafenib efficacy in xenografts embedded in old collagen [29].

A recurring observation in chronologically aged models is that multiple ECM variables often change simultaneously. As a result, these studies provide strong evidence that age-associated matrix states influence tumour behaviour, but they frequently cannot attribute the observed phenotype to a single causal ECM feature.

### 4.2 Defined age-associated ECM modifications

Defined age-associated ECM modifications provide greater mechanistic resolution by isolating individual ageing-related matrix features. Among these, collagen glycation offers a well-defined model of biochemical ECM modification. Dandia et al. showed that glucose-induced glycation of collagen I caused fibre bundling, increased pore size and storage modulus, and altered tumour-cell morphology and migration without affecting viability or proliferation. RAGE neutralisation supported a role for AGE–RAGE signalling [40]. This work showed that a single age-associated biochemical modification can affect multiple aspects of matrix function and cellular behaviour. Kumar et al. extended this approach to a perfused tumour–vessel chip. Methylglyoxal cross-linked collagen I, degraded laminin-rich basement membrane, and reduced adhesion of cancer and endothelial cells to both matrices. Exposure of the vascular channel to methylglyoxal increased cancer-cell intravasation [58]. Because methylglyoxal also induced endothelial senescence and shedding, this system could not separate matrix-dependent and endothelial effects.

In contrast, evidence from chronologically aged matrices shows that age-associated effects cannot be reduced to bulk stiffness. Aged tail-tendon collagen increased breast cancer invasion without measurable changes in bulk mechanics [39]. Collagen from old rats was markedly stiffer than young collagen [29], and aged mouse breast tissue was softer than young tissue before decellularisation but stiffer afterwards [26]. Receptor involvement also differed: RAGE blockade attenuated responses to glycated collagen [40], whereas RAGE did not mediate the proliferative response to naturally aged collagen, which reflected reduced DDR2 activation [25]. These findings suggest that ECM architecture, biochemical composition, and matrix organisation contribute to tumour phenotypes independently of bulk stiffness, and that a defined modification does not necessarily reproduce signalling of naturally aged matrix.

Defined age-associated ECM modification models therefore offer valuable mechanistic insight, although they replicate only specific aspects of the broader ageing phenotype.

### 4.3 Cell-mediated ECM remodelling

Cell-mediated remodelling (CMR) models incorporate active cellular contributions to ECM ageing. Unlike chronologically aged matrices or defined age-associated modifications, these approaches explicitly model ECM remodelling by living stromal populations. Senescence-associated remodelling (SAM) represents a specialised subtype of CMR in which senescence is the defined driver of matrix alteration [24], [61].

Ghosh et al. co-embedded breast cancer cells in collagen with mesenchymal stromal cells from a young donor made senescent by irradiation. Senescent cells increased collagen deposition, fibre bundling, and promoted spheroid growth and invasion. MMP inhibition abolished invasion in both senescent and control conditions, while LOX inhibition and periostin blockade only partially reduced the senescence-associated effect [61]. Kaur et al. showed that matrix deposited by fibroblasts from aged (>55 years) donors lacking HAPLN1 promoted melanoma invasion in 3D skin constructs and altered immune-cell motility. Restoring HAPLN1 reversed these effects [30].

These studies underscore the significance of cellular contributions to age-associated ECM remodelling and illustrate the difficulty of distinguishing ECM-mediated effects from ongoing stromal signalling. Consequently, CMR models, including SAM models, are especially valuable for investigating the ageing TME as an integrated biological system rather than isolating ECM-specific mechanisms.

### 4.4 What does the evidence demonstrate about age-aware modelling?

First, naturally aged extracellular matrices can alter multiple aspects of tumour behaviour across diverse cancer models. Enhanced invasion and motility are among the most consistently reported phenotypes [26], [29], [39]. Increased proliferation has been observed in prostate, fibrosarcoma, lung, and melanoma models [25], [27], [28], [29], [31], although not in every cell line [29], [39]. Emerging evidence also indicates that age-associated ECM states can influence therapeutic response, including altered sensitivity to erlotinib and vemurafenib [29], [31], [39]. Collectively, these findings support the conclusion that age-associated matrix states influence tumour behaviour rather than representing merely passive features of the TME.

Second, distinct modelling approaches generate different forms of evidence. Chronologically aged matrices provide strong evidence that age-associated ECM states matter biologically but offer limited ability to identify causal mechanisms because multiple variables change simultaneously [27], [39]. In contrast, defined modification models provide greater mechanistic resolution by isolating specific ageing-associated features, as shown by studies of collagen glycation and AGE-mediated signalling [40]. Cell-mediated remodelling models capture ageing as a dynamic biological process but introduce additional cellular and paracrine variables that complicate attribution of observed phenotypes [30], [61]. Third, no single ECM variable accounts for the observed phenotypes: biochemical modification, matrix remodelling or stromal-cell activity can each contribute [26], [30], [40], [61]. Age-associated tumour phenotypes do not consistently correlate with a single mechanical parameter such as stiffness. For example, enhanced invasion has been observed in aged matrices without measurable differences in bulk mechanical properties [39], while other aged matrices were substantially stiffer [29]. This indicates significant roles for architectural and biochemical remodelling.

Fourth, the current evidence base is narrow. All eleven studies model adult cancers, and none uses paediatric or developmental ECM as an experimental variable, although cancer can be diagnosed from birth [3] and developmental ECM differs from aged ECM in composition and organisation rather than representing an “unaged” state (Section 2.1). Six of the seven chronologically aged matrix studies use rodent tail-tendon collagen irrespective of the organ in which the tumour arises; only one uses organ-matched aged ECM [26], and only one uses matrix from aged human donors [30]. Age definitions also vary, from 2 to 6 versus 20–25 months in rodents to <45 versus >55 years in human donors, and only three studies include *in vivo* experiments [27], [29], [30]. No study yet combines aged ECM with aged stromal, immune and vascular components; the closest is a chip model in which methylglyoxal simultaneously modified the matrix and induced endothelial senescence [58].

Collectively, these observations support a framework-based interpretation of age-aware cancer models. The central consideration is not simply whether a model is aged, but rather which dimensions of ageing it replicates, how these dimensions have been validated, and the extent of mechanistic inference that can be drawn from the system. This perspective enables more meaningful comparisons among studies that might otherwise be grouped together under the broad designation of "aged ECM," despite representing fundamentally different experimental and biological conditions.

## 5 Design principles and future directions

The studies summarised in Table 3 show that age-associated ECM states affect tumour invasion, proliferation, signalling, and therapeutic response. However, experimental systems vary in how they define, model, and validate ageing. Progress in the field will require greater biological sophistication, improved reproducibility, clearer mechanistic interpretation, and standardised experimental methods.

### 5.1 Designing and validating age-aware 3D cancer models

A key challenge in age-aware cancer modelling is clearly defining and validating the ageing phenotype being reproduced. Chronological ageing, defined ECM modifications, cell-mediated ECM remodelling, and mixed multicomponent models are distinct approaches and should not be used interchangeably. The intended ECM state must be specified before model construction and independently verified using appropriate compositional, structural, biochemical, or mechanical measurements. Experimental interpretation should distinguish between the ageing intervention, the resulting ECM phenotype, and the downstream cancer response.

Mechanistic studies are most valuable when they extend beyond phenotypic observation to test whether specific ECM features are necessary or sufficient for the observed response. Examples include functional perturbation of LOX, hyaluronan supplementation, AGE–RAGE-associated signalling, DDR2/JAK2–ERK signalling, HAPLN1 restoration, and YAP/AXL-associated pathways [25], [26], [28], [29], [30], [31], [40]. These studies exemplify varying levels of mechanistic interrogation, from pathway association to direct functional perturbation.

To ensure reproducibility, studies must transparently report variables defining both the ageing model and cancer type (Box 1). At a minimum, this includes ECM source, donor age (if applicable), ageing intervention, matrix preparation method, validation measurements, cancer-cell characteristics, and controls. Consistent reporting of these parameters will facilitate cross-study comparisons and help identify reproducible age-associated effects. For rodent-derived matrices, donor age should be reported in months with the corresponding life stage, as mouse-to-human age relationships vary: about 2.6 mouse days equal one human year in adulthood, and mice are considered senescent from 18 months [66]. A single lifespan-wide conversion is therefore potentially misleading.

Box 1 **Reporting and Validation Criteria for Age-Aware ECM Models**

**Figure:**
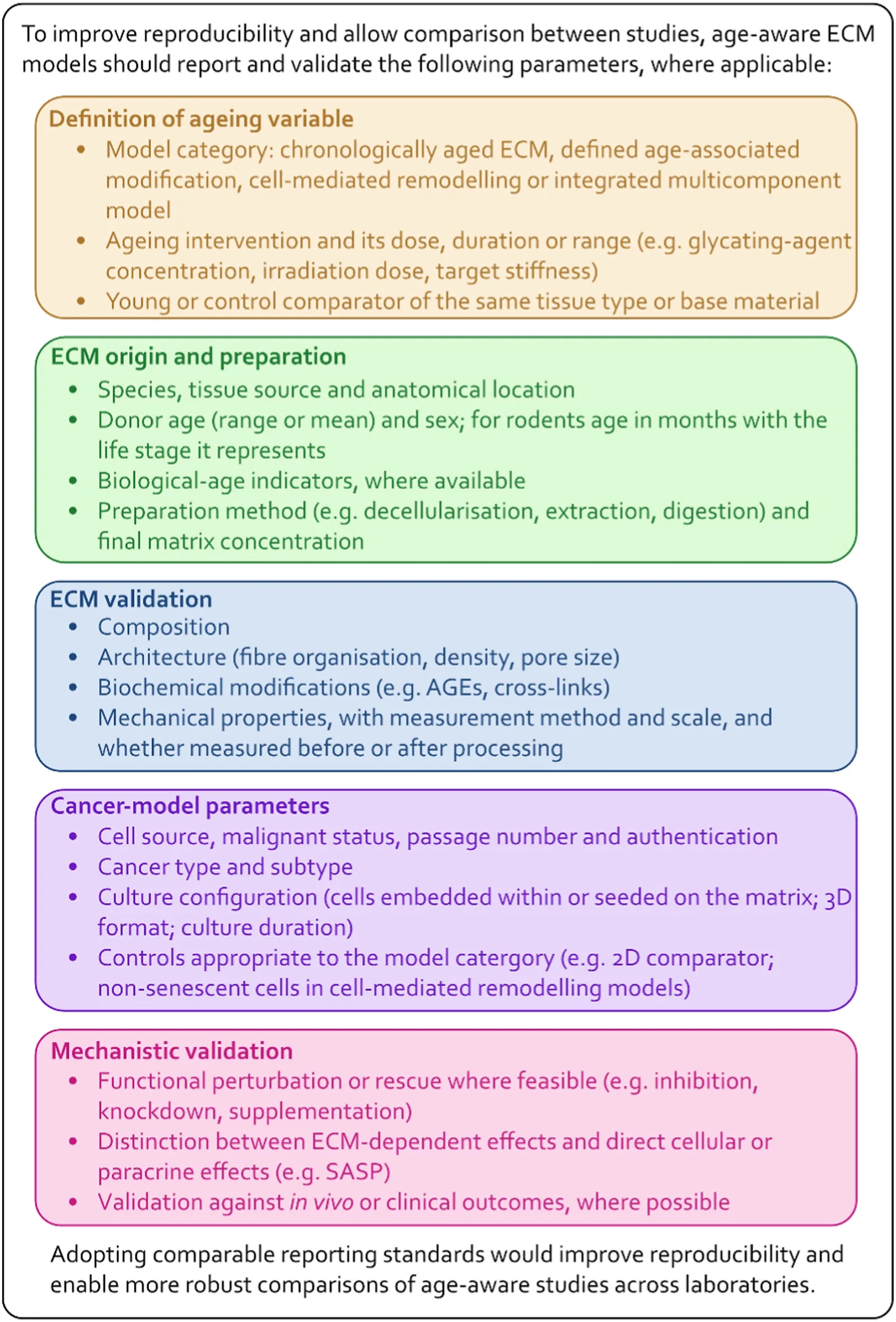

### 5.2 Integrating stromal, immune and vascular ageing

Ageing affects the TME beyond the ECM. Immune-cell composition, tissue distribution, and macrophage function all change with age [67], [68], [69], [70], [71]. Stromal and endothelial cells may also develop senescent phenotypes that remodel the matrix [24], [58], [61]. Future age-aware models should examine how ageing in these components interacts with ECM remodelling to influence tumour behaviour and therapeutic response.

This issue is especially relevant to cancer immunotherapy. Age-related changes in immune-cell composition and function have been observed in healthy individuals [67], [69], [70], [71]. In patients with solid tumours treated with immune-checkpoint inhibitors, those aged 65 or older had similar clinical outcomes to younger patients but showed reduced cytokine responses and smaller naïve T-cell pools [72]. Because the ECM regulates immune-cell migration, localisation, and tissue accessibility, interactions between ECM and immune ageing may influence therapeutic responses in ways not captured by current models.

Advanced 3D *in vitro* cancer-immune platforms offer a foundation for addressing these questions [63]. A promising strategy is to independently manipulate the age-associated state of different components within the same model. For example, combining young and aged ECMs with stromal or immune cells from various age groups allows systematic investigation of component-specific contributions. Modular approaches can help determine whether ageing in one component amplifies, reduces, or alters the effects of another.

Microfluidic and organ-on-chip technologies are well suited to these studies because they enable spatial organisation of tumour, stromal, vascular, and immune components, as well as controlled manipulation of age-associated variables [63], [64], [65].

### 5.3 Translation, standardisation and emerging opportunities

The translational value of age-aware cancer models depends on their ability to produce reproducible, biologically informative data. Current studies differ widely in ECM source, donor age, matrix processing, and validation methods, making direct comparison difficult. As the field advances, common reporting standards and validation criteria will be essential.

Another key challenge is distinguishing chronological age from biological ageing. Individuals of the same chronological age can differ significantly in tissue remodelling, matrix composition, and cellular ageing. Including molecular and biochemical indicators of biological ageing alongside donor age may improve interpretation of age-associated phenotypes and increase the translational relevance of age-aware cancer models [73], [74].

Patient-derived systems offer significant opportunities for future development. Organoids, engineered tissues, and related patient-specific models can incorporate both chronological and biological ageing variables while preserving clinically relevant tumour heterogeneity [75], [76]. However, chronological age alone does not necessarily reflect the biological state of the TME. Direct assessment of ECM and cellular ageing, as well as cancer type features, remains essential.

Age-aware three-dimensional models are also pertinent to replacing animal experiments, as outlined in the 3Rs principle of EU Directive 2010/63/EU and in new approach methodology (NAM) programmes [77], [78], [79]. Several challenges must be addressed before these models can reduce reliance on aged animals. First, current *in vitro* systems do not replicate systemic features of ageing, such as immune ageing and chronic low-grade inflammation [67], [68], [69], [70], [71]. Second, few studies validate *in vitro* age-associated effects against *in vivo* or clinical outcomes; among the studies in Table 3, only three include *in vivo* experiments [27], [29], [30]. Third, aged human tissue is scarce, and most aged matrices come from rodents, creating a species gap. Fourth, heterogeneity in ECM source, preparation, and validation limits reproducibility. Finally, regulatory acceptance of data from new approach methodologies faces scientific, technical, legislative, economic, and cultural barriers, as analysed for chemical safety assessment [79]. Standardised reporting (Box 1) and high-throughput formats, such as the 720-compound screen conducted in young and aged matrices [39], may help address these challenges.

The lack of paediatric models (Section 4.4) presents a distinct opportunity. Paediatric solid tumours develop in tissues with unique ECM characteristics [44], [45], [46], [47], [48], [49], [50], [51], [52], [53] and neuroblastoma cells exhibit different invasion strategies depending on matrix context [53]. In neuroblastoma, stromal collagen XI expression correlates with advanced stage, relapse, poorer survival, and diagnosis at 18 months of age or older [48], indicating that ECM state may vary with age even within childhood. Paediatric tumouroids bioprinted in ECM-mimetic hydrogels, designed from matrix gene expression in neuroblastoma and sarcoma patient cohorts, already support high-throughput drug screening [80], providing platforms to introduce developmental ECM states. Incorporating developmental ECM into three-dimensional platforms would enable the study of age across the full lifespan rather than focusing solely on older ages. However, access to paediatric material remains a practical and ethical challenge, as international guidance on paediatric biobanking is largely non-binding and inconsistent, including on reconsent when participants reach adulthood [81].

Advances in bioprinting, biomaterials engineering, and organ-on-chip technologies offer greater control over matrix composition, architecture, and cellular organisation [9], [64], [65]. These platforms support modular approaches where individual age-associated features can be introduced, validated, and systematically assessed, enabling more precise investigation of causality and tumour–microenvironment interactions.

Age-aware systems will generate increasingly complex datasets that may benefit from computational approaches integrating matrix composition, imaging, biomechanics, signalling, and treatment response, as long as these models are based on experimentally validated biological systems [11], [82], [83].

Greater biological complexity should not be an end in itself. The most effective model is the simplest experimentally validated system that reproduces the ageing-associated features relevant to the specific biological question.

## 6 Conclusions

Age-associated ECM remodelling is a multidimensional process that involves changes in composition, architecture, biochemical modification, mechanics, and turnover. These features are interconnected but not interchangeable, and current evidence does not support using a single molecular or mechanical surrogate for an aged ECM.

Current evidence demonstrates that age-associated ECM states influence tumour-cell invasion, proliferation, signalling, and therapeutic response across multiple cancer models. However, age-aware systems vary substantially in the dimensions of ageing they reproduce and the strength of mechanistic inference they allow. Chronologically aged matrices offer the highest biological fidelity to naturally occurring ageing, while defined modifications provide greater experimental control and mechanistic resolution. Cell-mediated systems capture ageing as an active cellular process but introduce additional variables.

The principal implication is that age-aware ECM models should be selected and interpreted based on the specific ageing-associated features they reproduce, the extent of experimental validation, and the required level of causal inference. Rather than maximising model complexity, future studies should align biological fidelity and experimental control with the research question.

## CRediT authorship contribution statement

**Vanessa Farkas:** Writing – review & editing, Writing – original draft, Visualization, Investigation, Funding acquisition. **Anusha Ashokan:** Writing – review & editing, Writing – original draft, Investigation, Funding acquisition. **Ronja Struck:** Writing – review & editing, Investigation. **Hannah O'Neill:** Writing – original draft, Investigation. **Fabio Quondamatteo:** Writing – review & editing, Conceptualization. **Olga Piskareva:** Writing – review & editing, Writing – original draft, Visualization, Supervision, Resources, Project administration, Investigation, Funding acquisition, Conceptualization.

## Declaration of generative AI and AI-assisted technologies in the manuscript preparation process

During the preparation of this work, the authors used Elicit, Claude and Grammarly to compile research papers, structure the manuscript, verify consistency of statements and references, and improve Grammar, sentence structure, and story flow. The authors reviewed and edited the output as needed and take full responsibility for the content of the published article. All illustrations were created using Adobe Photoshop.

## Declaration of competing interest

The authors declare that they have no known competing financial interests or personal relationships that could have appeared to influence the work reported in this paper.

## Data Availability

No data was used for the research described in the article.
